# Limited Dengue Virus Replication in Field-Collected *Aedes aegypti* Mosquitoes Infected with *Wolbachia*

**DOI:** 10.1371/journal.pntd.0002688

**Published:** 2014-02-20

**Authors:** Francesca D. Frentiu, Tasnim Zakir, Thomas Walker, Jean Popovici, Alyssa T. Pyke, Andrew van den Hurk, Elizabeth A. McGraw, Scott L. O'Neill

**Affiliations:** 1 School of Biological Sciences, Monash University, Clayton, Victoria, Australia; 2 Public Health Virology, Forensic and Scientific Services, Department of Health, Archerfield, Queensland, Australia; 3 Institute for Molecular Biosciences, The University of Queensland, St. Lucia, Queensland, Australia; United States Army Medical Research Institute of Infectious Diseases, United States of America

## Abstract

**Introduction:**

Dengue is one of the most widespread mosquito-borne diseases in the world. The causative agent, dengue virus (DENV), is primarily transmitted by the mosquito *Aedes aegypti*, a species that has proved difficult to control using conventional methods. The discovery that *A. aegypti* transinfected with the *w*Mel strain of *Wolbachia* showed limited DENV replication led to trial field releases of these mosquitoes in Cairns, Australia as a biocontrol strategy for the virus.

**Methodology/Principal Findings:**

Field collected *w*Mel mosquitoes that were challenged with three DENV serotypes displayed limited rates of body infection, viral replication and dissemination to the head compared to uninfected controls. Rates of dengue infection, replication and dissemination in field *w*Mel mosquitoes were similar to those observed in the original transinfected *w*Mel line that had been maintained in the laboratory. We found that *w*Mel was distributed in similar body tissues in field mosquitoes as in laboratory ones, but, at seven days following blood-feeding, *w*Mel densities increased to a greater extent in field mosquitoes.

**Conclusions/Significance:**

Our results indicate that virus-blocking is likely to persist in *Wolbachia*-infected mosquitoes after their release and establishment in wild populations, suggesting that *Wolbachia* biocontrol may be a successful strategy for reducing dengue transmission in the field.

## Author Summary

Almost half of the world's population is at risk of contracting dengue virus, particularly in the tropics and sub-tropics. The virus is transmitted by the mosquito *Aedes aegypti*, a cosmopolitan species that has proved difficult to control using traditional methods. A new biocontrol strategy has been developed involving the release of mosquitoes infected with *Wolbachia* bacteria. Mosquitoes with the *w*Mel strain of *Wolbachia* show dramatically reduced replication and transmission of dengue virus in laboratory trials. Although promising, the utility of *Wolbachia* biocontrol depends on field *w*Mel-infected mosquitoes retaining the phenotype of reduced viral replication. Mosquitoes with *w*Mel were released in the field in Cairns, Australia in early 2011. We provide evidence that, one year later, field collected *w*Mel mosquitoes showed reduced dengue virus replication in the body and limited dissemination to the head compared to controls. *Wolbachia* numbers in mosquitoes increased following blood meals, which may further decrease viral replication if the insects feed frequently. Our results indicate that *Wolbachia*-mediated dengue interference is sustained in field populations and shows no sign of attenuation after one year of deployment.

## Introduction

Dengue is one of the most common and widespread vector-borne diseases in the world, with up to 380 million infections estimated to occur annually [1]. The causative agent, dengue virus (DENV), has expanded its geographic range in the last two decades, with more than 100 countries now affected. Infection with DENV leads primarily to self-limiting fevers but recent decades have seen a marked increase in severe dengue, with manifestations such as hypovolemic shock and hemorrhage [2]. DENV is transmitted primarily by the mosquito vector *Aedes aegypti* and, to a lesser extent, by its congener *A. albopictus*. In the absence of an effective vaccine [3] and/or antivirals, prevention of dengue transmission relies primarily on control of mosquito vectors. The failure to prevent the global spread of dengue, increasing insecticide resistance in mosquito populations and subsequent escalating costs of insecticide-based programs, as well as environmental concern over the impact of these chemicals, have spurred the development of novel, inexpensive and green vector control methods [4], [5].

The transinfection of vector mosquitoes with the bacterium *Wolbachia pipientis* has emerged as a promising method for the control of dengue. *Wolbachia* is the most common endosymbiont of insects, thought to infect up to 40% of arthropod species [6]. *A. aegypti* stably transinfected with different strains of *Wolbachia* show reduced replication and transmission of DENV [7]–[9]. An additional advantage of using *Wolbachia* for biocontrol of DENV is the ability of the bacterium to propagate through a population by inducing cytoplasmic incompatibility (CI) in its host [10]. CI confers a fitness advantage to *Wolbachia*-infected females that allows these maternally transmitted bacteria to spread unaided through a population [10]. The use of *Wolbachia* provides a means of biocontrol that is both pesticide-free and poses minimal environmental safety concerns [11].

In laboratory trials, mosquitoes with the *w*Mel strain of *Wolbachia* showed both blocking of DENV transmission and minimal fitness effects due to infection with the bacterium [9]. In addition, *w*Mel rapidly invaded wildtype mosquito populations in semi-field cage experiments due to CI and minimal fitness costs [9]. The results facilitated the field release of *w*Mel-infected mosquitoes in two suburbs of Cairns, Queensland, Australia [12]. Within a short period, the frequency of *w*Mel reached fixation in the two suburbs [12] and has remained established at both sites.

The persistence of the viral-blocking phenotype in field populations is fundamental to the utility of releases of *Wolbachia*-infected mosquitoes. The mechanisms that underpin viral interference are poorly understood but may be related to the density of *Wolbachia* [13], [14], immune pre-activation [7], [8], [15], intra-host competition for cellular resources [16], [17] or suppression of host cellular factors that are upregulated during viral infection [18]. The density of *Wolbachia* may decrease after several generations, as happened following the transinfection of the virulent strain of *w*MelPop into the novel host *Drosophila simulans* [19]. *Wolbachia* infection frequencies and associated CI effects may also be significantly lower in nature than observed in the lab, as observed in *Drosophila simulans* [20]. However, the *w*Mel strain is avirulent and has limited negative effects on mosquito fitness in the laboratory [9], suggesting that the density of the *w*Mel strain may remain stable over time. Protection against RNA virus-induced mortality was in fact first observed in the long term, evolutionarily stable association between *w*Mel and its *Drosophila melanogaster* host [21].

Here, we investigated the extent of virus blocking in field *w*Mel-infected *A. aegypti*, one year following field release, using three serotypes of DENV. We found limited replication and dissemination of DENV in field *w*Mel mosquitoes, indicating stability of the viral-blocking phenotype in wild *Wolbachia*-infected mosquitoes. The extent of virus blocking was similar in field mosquitoes compared to the original, *w*Mel-infected, outcrossed lab line used for release. Interestingly, the density of *Wolbachia* increased following blood feeding and to a greater extent in field versus lab *w*Mel-infected mosquitoes. We suggest that if the viral blocking effect of field *w*Mel is dependent on *Wolbachia* density, repeated blood feeding on human hosts might amplify this effect. Our results reinforce the utility of *Wolbachia*-based technology for biocontrol of dengue.

## Methods

### Ethics statement

Blood feeding of mosquito colonies using human volunteers was performed in accordance to Monash University Human Research Ethics Committee permit CF11/0766-2011000387. Written informed consent was obtained from all volunteers who participated in the study. Dengue viremic plasma was obtained from patients enrolled in a prospective study at the Hospital for Tropical Diseases, Ho Chi Minh City, Vietnam. All patients provided written consent to participate in the study. The study protocols relevant to this work, including vector competence experiments, were reviewed and approved by the Scientific and Ethical Committee of the Hospital for Tropical Diseases (CS/ND/09/24) and the Oxford Tropical Research Ethical Committee (OxTREC 20-09). The inclusion criteria were: a) adult patients (≥15 years of age), with ≤72 hours of fever and suspected of having dengue based on clinical symptoms, b) a positive NS1 Rapid test and c) written informed consent. All plasma samples were anonymized (samples were identified using numbers only) prior to experiments.

### Mosquito colony establishment and maintenance

Mosquito eggs were collected in January 2012 from ovitraps placed inside the *Wolbachia* release zone in the Cairns suburbs of Yorkey's Knob and Gordonvale and outside, in Edge Hill, Whitfield, Edmonton and Bentley Park. Eggs collected from outside the *Wolbachia* release zone were *Wolbachia*-uninfected. Eggs on ovistrips were allowed to hatch and larvae reared in water supplemented with fish food pellets (Tetramin, Tetra). Fourth instar larvae were identified as *A. aegypti* based on specific morphological characters. Adults (F_0_) emerged in cages of approximately 450 individuals and were allowed to feed on 10% sucrose *ad libitum*. Five to seven day old females were allowed to feed on human volunteers and eggs were collected from several gonotrophic cycles. F_1_ adults hatched from eggs obtained in the first gonotrophic cycle were used in vector competence experiments. The *w*Mel-infected field mosquito line and its uninfected counterpart (derived from *Wolbachia*-uninfected eggs) were denoted *w*Mel.F and wildtype, respectively. The original laboratory-reared, outcrossed *w*Mel-infected MGYP2.out line [9] was used in some experiments. All mosquito colonies were kept at 26°C under a 12L∶12D light cycle and 60% relative humidity.

### Virus strains

Mosquitoes were challenged in vector competence experiments with virus strains belonging to DENV serotypes 1–3, using virus grown in cell culture and viremic plasma from human patients. DENV-2 strain 92T and DENV-3 strain Cairns 2008 (both isolated from outbreaks in north Queensland, Australia in 1992 and 2008, respectively) were grown in C6/36 cells and harvested and titered as described previously [13]. Virus was aliquoted in single-use 1 mL lots and stored at −80°C.

### Vector competence experiments

Two separate vector competence experiments were carried out to determine if DENV could replicate and disseminate in field *w*Mel-infected mosquitoes. For both experiments, female mosquitoes (5–7 days old) were allowed to feed on viremic blood meals contained in a membrane feeder with sheep intestine as the membrane. Virus was mixed with defibrinated sheep blood to obtain final bloodmeal titers (see below). Mosquitoes were allowed to feed for 1 hour, with engorged females separated from unfed ones the next day. Females were kept in plastic cups at a density of 10–12 individuals/cup and allowed access to 10% sucrose *ad libitum*. Females were killed under CO_2_ at either 7 or 14 days post infection (p.i.), immediately frozen in dry ice and stored at −80°C until further processing.

In the first experiment, field *w*Mel and uninfected mosquitoes were challenged with two viremic plasma samples from Vietnam, DENV-1 – P249 (final titer 7.38E+08 genomic copies/mL) and DENV-2 – P410 (final titer 1.12E+09 genomic copies/mL), as well as a cell-culture grown virus isolated in Australia, DENV-2 – 92T (9.30E+09 copies/mL) as a control. In the second experiment, the field *w*Mel-infected and two control lines, MGYP2.out [9] and field *Wolbachia*-uninfected wildtype, were challenged with a viremic human plasma sample from Vietnam, DENV-1-P307 (2.46E+11 copies/mL), and two virus strains isolated in Australia, DENV-2-92T (9.30E+09 copies/mL) and DENV-3-Cairns 2008 (3.58E+09 copies/mL). Human viremic plasmas underwent a single freeze-thaw cycle before use in vector competence experiments.

### RNA extraction and qRT-PCR for DENV

RNA was extracted from mosquito bodies using Trizol reagent (Invitrogen), and from heads using the QIAamp viral RNA mini kit (Qiagen), following homogenization of tissues with 3 mm glass beads in a Beadbeater. A higher yield of total RNA was obtained on average from head samples using the QIAamp viral RNA mini kit versus Trizol (F. Frentiu, unpublished data). For mosquitoes challenged with Vietnamese viremic plasmas, virus genome copies were estimated by qRT-PCR using FAM-labeled DENV-1 and DENV-2 hydrolysis probe sequences and standard curves from reference [22]. Virus copies in mosquitoes challenged with DENV-2-92T and DENV-3-Cairns 2008 were estimated by qRT-PCR, using hydrolysis probes specific to the 3′UTR region. Primer sequences were F: 5′-AAGGACTAGAGGTTAGAGGAGACCC-3′ and R: 5′-CGTTCTGTGCCTGGAATGATG-3′, with probe sequence: 5′- FAM- AACAGCATATTGACGCTGGGAGAGACCAGA-BHQ1-3′. Reactions were performed with the SuperScript® III Platinum® One-Step qRT-PCR kit (Invitrogen) and contained 5 µL of RNA template, 5 µM each of probe and forward and reverse primers, buffer and enzyme as per kit instructions, in a total volume of 20 µL. For head qPCRs, 10 µL of RNA template was used, with water adjusted accordingly. The number of DENV copies was calculated following a standard curve for DENV 3′UTR, constructed as in [8]. All reactions were performed using a LightCycler480 Instrument (Roche) with the following run conditions: 50°C for 15 min, 95°C for 2 min, followed by 45 amplification cycles of 95°C for 15 s, 60°C for 30 s and a final cooling step of 40°C for 10 s.

Reactions were run in duplicate and samples where DENV failed to amplify in at least one replicate were classified as zero. Only samples where DENV amplified in both technical replicates and the amount of copies extrapolated by the LightCycler software was above the lower bound of the standard curve (limit of detection) were included in the analysis. All mosquitoes from field and lab *w*Mel-infected lines that showed DENV breakthrough were tested for the presence of *Wolbachia* using IS5 repeat primers specific to the *w*Mel and *w*MelPop strains [23]. Only one sample each from the field and lab *w*Mel-infected mosquitoes was negative for *Wolbachia*. These samples were excluded from further analysis.

### DNA extraction and quantification of *Wolbachia* density

The densities of *Wolbachia* were compared between field and lab strains of *w*Mel-infected mosquitoes in a separate experiment. Five to seven-day old females from each line were fed on a mix of DENV-3 – Cairns 2008 and sheep blood and collected at 7 and 14 days post infection (as detailed above) for genomic DNA extraction. Control non-blood fed females from each line were maintained in parallel and collected at the same time points. Genomic DNA was extracted using the DNAEasy Blood and Tissue kit (Qiagen) as per the manufacturer's instructions. A multiplex qPCR amplifying the target *Wolbachia*-specific *wsp* and mosquito housekeeping *Rp*S*17* [24] genes was performed (*wsp* F: 5′-CATTGGTGTTGGTGTTGGTG-3′, R: 5′-ACACCAGCTTTTACTTGACCAG-3′, probe: 5′-HEX-TCCTTTGGAACCCGCTGTGAATGA-BHQ1-3′; *Rp*S*17* F: 5′-TCCGTGGTATCTCCATCAAGC-3′, R: 5′-CACTTCCGGCACGTAGTTGTC-3′, probe: 5′-FAM-CAGGAGGAGGAACGTGAGCGCAG-BHQ1-3′).The *Rp*S*17* housekeeping gene was used to normalize *wsp* gene copies. qPCR reactions were performed in 10 µL total volume containing 1× Lightcycler 480 Probes Master reaction mix, 5 µM each of *wsp* primers and probe, 2.5 µM each of *Rp*S*17* primers and probe and 1 µL of DNA template. Cycling was performed using a LightCycler480 Instrument (Roche), with 1 cycle at 95°C for 5 min, followed by 45 amplification cycles of 95°C for 10 s, 60°C for 15 s, 72°C for 1 s, and a final cooling cycle of 40°C for 10 s. Target to housekeeping gene ratios were calculated using the Relative Quantification algorithm in the Lightcycler 480 software (Roche).

### Fluorescence in-situ hybridization (FISH)

Tissue localization of *w*Mel in field *w*Mel.F and lab MGYP2.out mosquitoes was visualized using FISH. Females were collected under CO_2_ and immediately placed overnight in 4% paraformaldehyde at 4°C with their wings and legs removed. Paraffin-embedded mosquitoes were sectioned in 8 µM thin slices. Slides were de-paraffinated in 100% xylene, rehydrated in an ethanol series and hybridized overnight at 37°C in a buffer containing *Wolbachia*-specific W2 and W3 probes [8]. Post-hybridization processing followed [8]. Slides were mounted using an antifade reagent (Prolong Gold, Invitrogen) and viewed with a Zeiss Axio Imager II epifluorescence microscope equipped with an Axiocam camera, using the same exposure conditions for each filter channel.

### Statistical analysis

Differences between mosquito lines in DENV infection rates for both vector competence experiments were analyzed using pairwise Fisher's exact tests. P-values were adjusted for multiple comparisons for each day of sampling within each experiment using the Holm method [25], with values <0.05 considered significant. In experiment 1, differences in median DENV copy numbers between lines were analyzed using Mann-Whitney U tests. In experiment 2, differences among the three lines in copies of each virus were analyzed using Kruskal-Wallis tests, with Dunn's post-hoc multiple comparison tests. Last, we tested for significant differences in *Wolbachia* density between MGYP2.out and *w*Mel.F mosquitoes using Mann-Whitney U tests. All analyses were performed in R [26] and GraphPad Prism v. 6 (GraphPad Software, San Diego, California USA).

## Results

### Limited DENV infection and replication in field *w*Mel-infected mosquitoes

**Figure 1:**
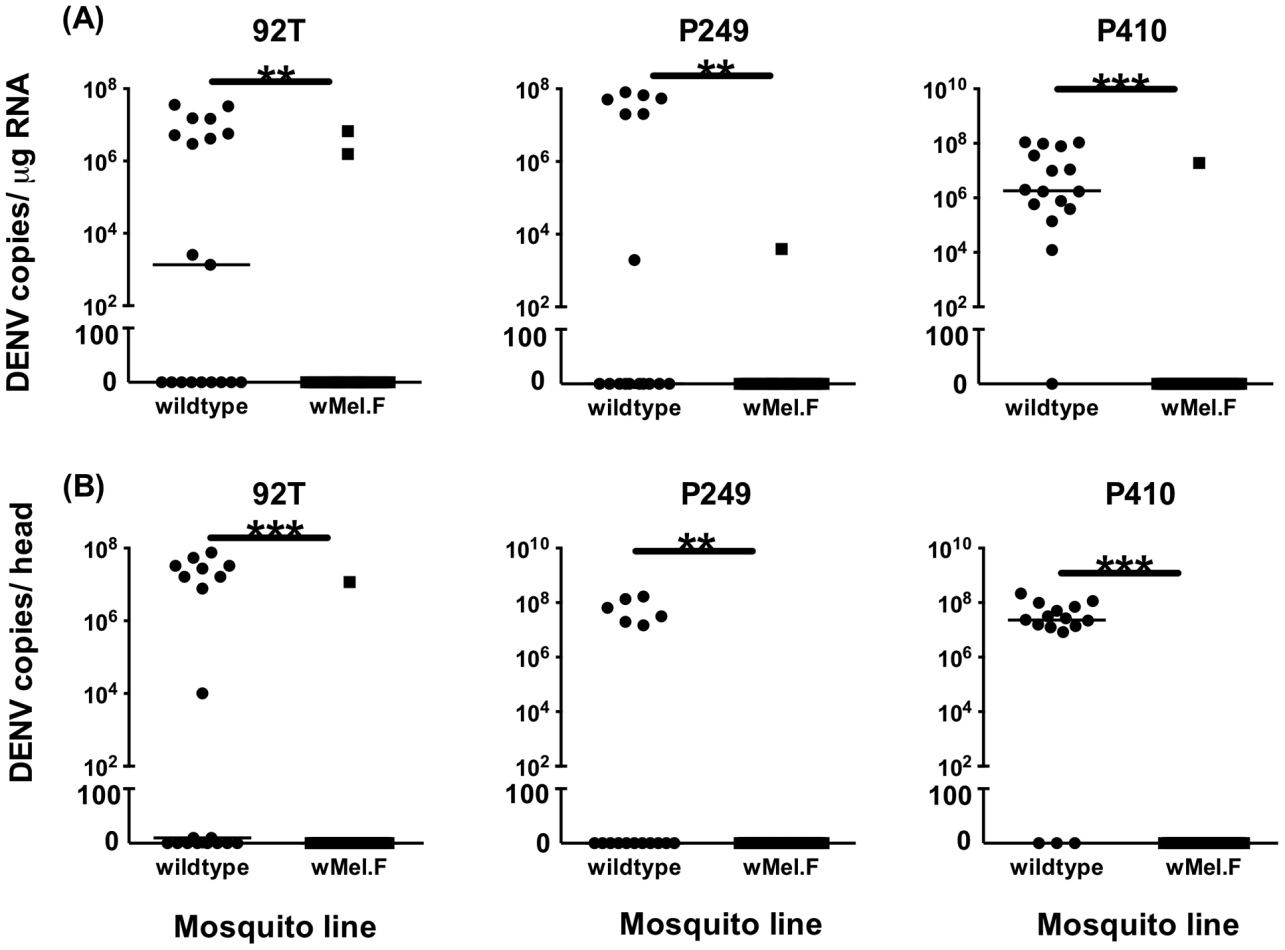
Experiment 1: DENV replication in wildtype and field-released (*w*Mel.F) *A. aegypti*. DENV replication in bodies (A) and heads (B) of mosquitoes challenged with three strains (DENV2-92T, DENV1-P249, DENV2-P410), assayed at 14 days post-infection. DENV levels determined using one-step qRT-PCR and expressed as copies per 1 µg of total RNA. Bars denote medians. P<0.05 (*), P<0.01 (**), P<0.001 (***). Each point represents an individual mosquito.

We conducted two independent experiments to assess rates of DENV infection and replication in wildtype and *w*Mel-infected field release mosquitoes. In experiment 1, at day 7 p.i., lower rates of body and head infection were detected in field *w*Mel mosquitoes compared to wildtype for the two DENV-1 and DENV-2 viremic plasma samples and cell culture DENV-2-92T virus strains (**Table 1**). However, only for DENV-2 strain P410, a viremic plasma sample, was there a statistically significant difference between the two mosquito lines (**Table 1**). At day 14 p.i., rates of body and head infection were significantly lower in field *w*Mel compared to wildtype mosquitoes for all three DENV strains, with a stronger effect in dissemination to heads (**Table 1**). The highest observed dissemination rate in *w*Mel.F heads was a low 6%, compared to 62% in wildtype heads. DENV genome copy titers in heads and bodies were uniformly higher for all strains in wildtype mosquitoes compared to respective *w*Mel.F samples at day 14 p.i. (**Figure 1**). For example, titers in both bodies and heads typically reached 1×10^8^ copies for all virus strains in wildtype individuals. By contrast, most *w*Mel.F individuals showed an absence of DENV replication (**Figure 1**). A similar difference in virus titers was present at day 7 p.i., but to a lesser extent because of low infection rates (**Figure S1**).

**Table 1:** Rates of infection (%) for three DENV strains between field *Wolbachia*-infected (*w*Mel.F) and uninfected (wildtype) mosquito lines at days 7 and 14 p.i. (experiment 1).

	Body infection (*N*)		Head infection (*N*)	
	wildtype	*w*Mel.F	wildtype	*w*Mel.F
*day 7 p.i.*				
DENV1 – P249	44 (18)	19 (16)	44 (18)	6 (16)
DENV2 – 92T	26 (21)	0 (18)	10 (21)	0 (17)
DENV2 – P410	79 (14)	6 (31)***	62 (13)	3 (31)***
*day 14 p.i.*				
DENV1 – P249	41 (17)	4 (25)*	35 (17)	0 (25)***
DENV2 – 92T	53 (19)	7 (28)*	47 (19)	4 (28)***
DENV2 – P410	94 (16)	3 (30)*	62 (13)	3 (30)***

Adjusted Fisher's exact test p-values<0.05 (*), <0.001 (***).

We next investigated whether vector competence was similar in field *w*Mel-infected *A. aegypti* compared to the original *w*Mel-infected line that had been maintained in the lab with recurrent outbreeding [9]. In experiment 2, we estimated DENV infection rates and replication titers for three virus strains in wildtype, *w*Mel.F and MGYP2.out mosquitoes. We tested for statistically significant differences in infection rates only between wildtypes and *w*Mel.F, and between *w*Mel.F and MGYP2.out mosquitoes (**Table 2**). At day 7 p.i., significantly lower body infection rates were found in *w*Mel.F mosquitoes versus wildtypes for DENV-2-92T and DENV-3-Cairns08 strains (**Table 2**). However, rates of infection across all mosquito lines and all viruses were low in general, resulting in limited power for robust statistical tests. At day 14 p.i., significantly different infection rates between wildtypes and *w*Mel.F mosquitoes were found for both bodies and heads across all DENV strains (**Table 2**). For both experiments 1 and 2, dissemination of all virus strains by day 14 p.i. was dramatically lower in field *w*Mel mosquitoes compared to wildtypes. There were no significant differences in infection rates between *w*Mel.F and MGYP2.out mosquitoes across either day post-infection.

**Table 2:** Rates of infection (%) for three DENV strains among field (*w*Mel.F) and laboratory *Wolbachia*-infected (MGYP2.out) and uninfected (wildtype) mosquito lines at days 7 and 14 post-infection (p.i.) (experiment 2).

	Body infection (*N*)			Head infection (*N*)		
	wildtype	*w*Mel.F	MGYP2.out	wildtype	*w*Mel.F	MGYP2.out
*day 7 p.i.*						
DENV1 – P307	23(13)	12 (17)	10 (21)	8 (13)	6 (17)	5 (21)
DENV2 – 92T	54 (13)	0 (16)*	13 (15)	8 (12)	0 (16)	7 (15)
DENV3 – Cairns08	58 (12)	6 (17)*	14 (14)	25 (12)	0 (17)	7 (14)
*day 14 p.i.*						
DENV1 – P307	65 (17)	15 (20)*	41 (17)	65 (17)	5 (20)***	29 (17)
DENV2 – 92T	77 (13)	12 (17)*	13 (15)	69 (13)	6 (17)***	7 (14)
DENV3 – Cairns08	92 (13)	6 (17)***	9 (22)	77 (13)	6 (17)***	0 (22)

Adjusted Fisher's exact test p-values<0.05 (*), <0.001 (***). P-values shown refer to comparisons between wildtype and *w*Mel.F mosquitoes.

**Figure 2:**
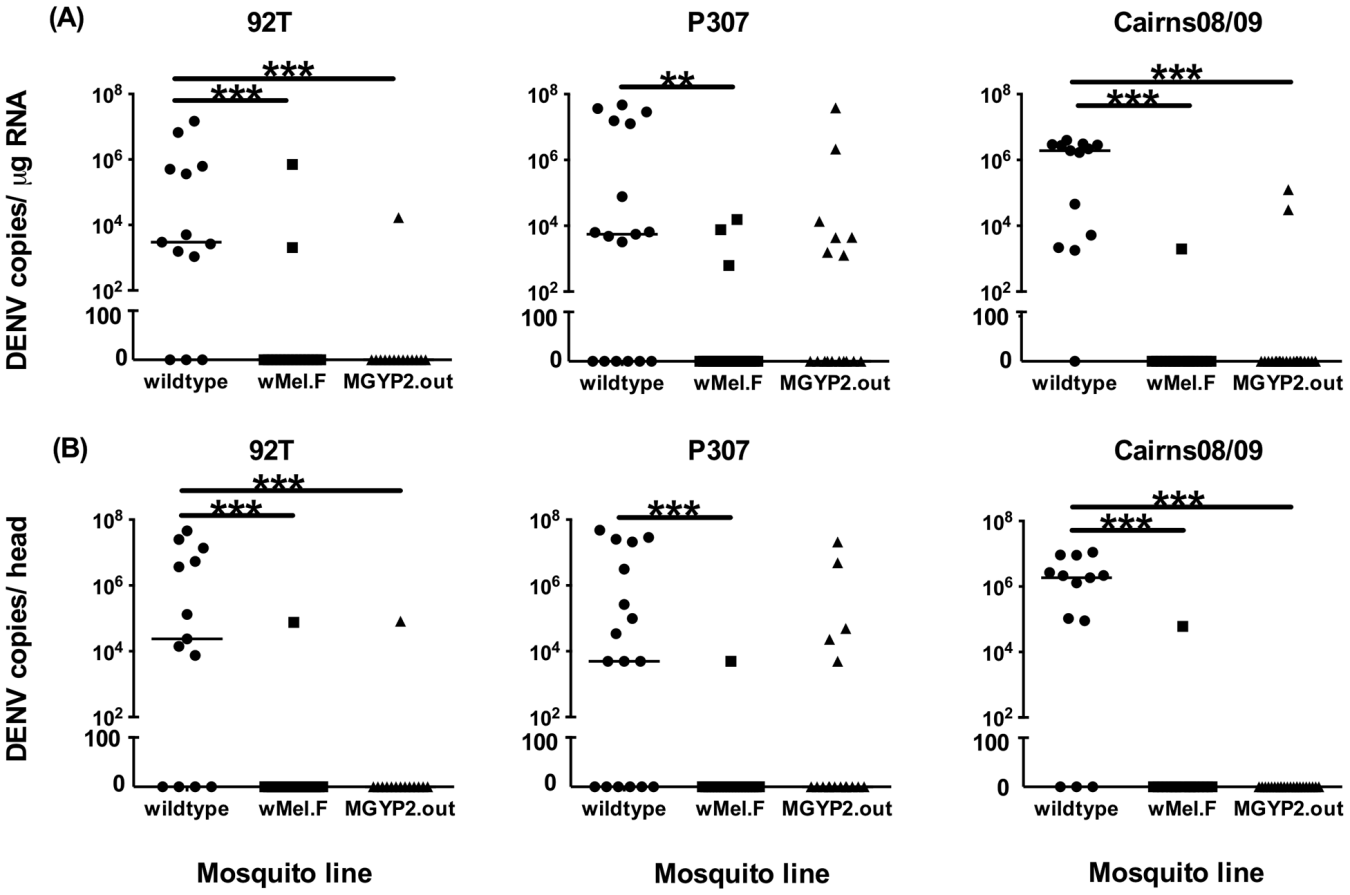
Experiment 2: DENV replication in wildtype, outbred laboratory *w*Mel (MGYP2.out) and field-released *w*Mel (*w*Mel.F) *A. aegypti*. DENV replication in bodies (A) and heads (B) of mosquitoes challenged with three strains (DENV2-92T, DENV1-P307, DENV3-Cairns08/09), assayed at 14 days post-infection. DENV levels determined using one-step qRT-PCR and expressed as copies per 1 µg of total RNA. Bars denote medians. P<0.05 (*), P<0.01 (**), P<0.001 (***). Each point represents an individual mosquito.

DENV titers were significantly lower across all virus strains in both heads and bodies in field *w*Mel mosquitoes compared to wildtypes, at day 14 post-infection (**Figure 2**). A similar pattern was observed at day 7 post-infection, although only for bodies and the strains DENV-2-92T and DENV-2-Cairns08/09 (**Figure S2**). At day 14, virus titers in wildtype mosquitoes ranged from below the limit of detection to 10^8^ copies/µg of RNA whereas virus was observed only in a few instances in field *w*Mel. Only in one field *w*Mel individual was the maximum number of DENV copies observed (**Figure 2**, strain 92T body and heads panels). Overall, the results indicate that when breakthrough virus occurs in *w*Mel mosquitoes, viral titers are most likely to be lower than those observed in wildtypes.

### *Wolbachia* tissue tropism and density in field mosquitoes

**Figure 3:**
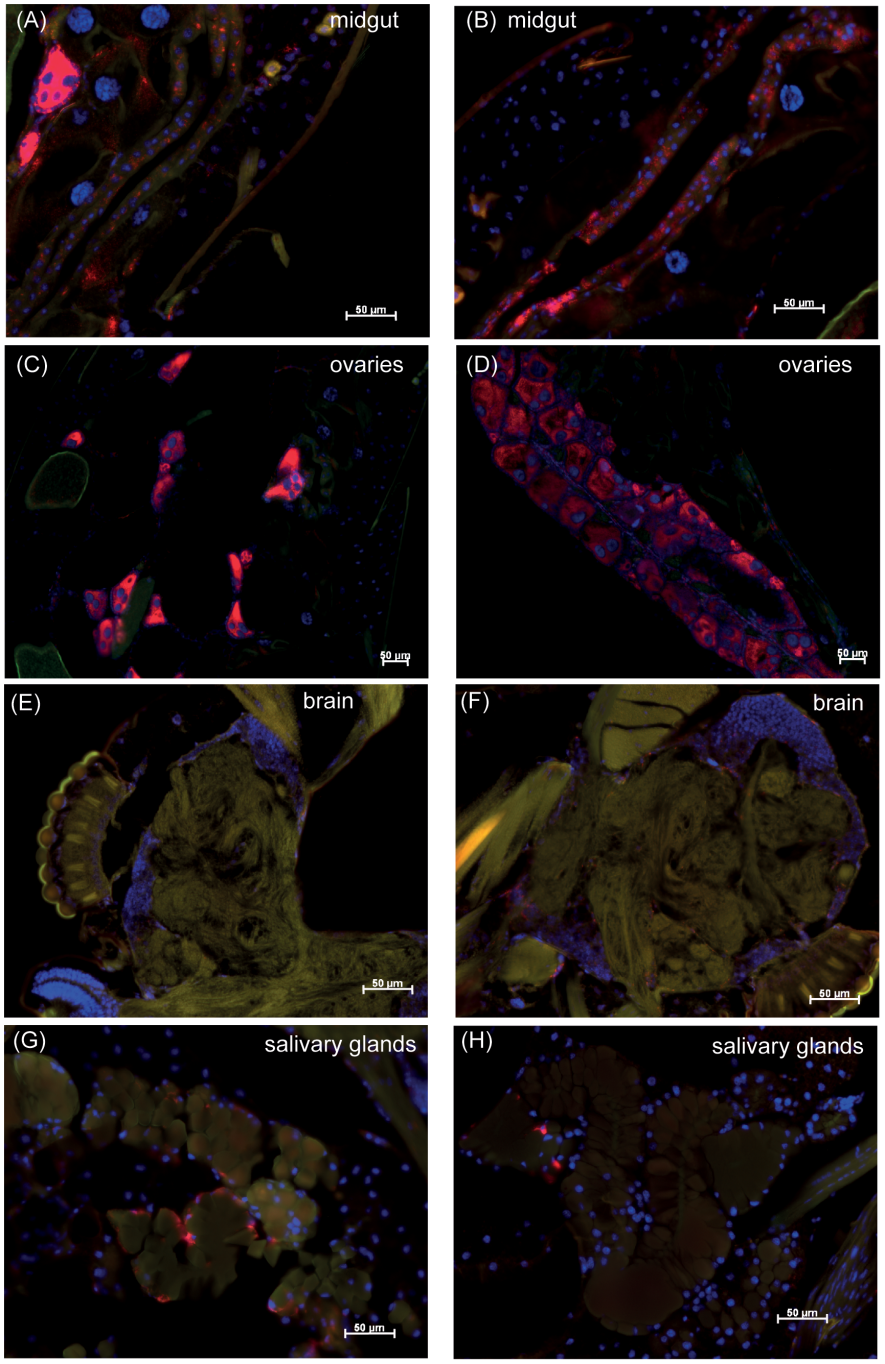
Localization of *Wolbachia* in different *A. aegypti* tissues visualized using FISH. Outbred laboratory *w*Mel (MGYP2.out) (A, C, G, E) and field-released *w*Mel (*w*Mel.F) (B, D, F, H) mosquitoes at day 7 post DENV infection. *Wolbachia* stained in red (Alexa 594) and cell nuclei in blue (DAPI). Images are representative of 4–5 mosquitoes per line. Bars represent 50 µM scale.

We next investigated whether *Wolbachia* tissue tropism and density had changed significantly in field *w*Mel mosquitoes since release in 2011. Using FISH, we found that *Wolbachia* was distributed in the same tissues in field mosquitoes and in the original *w*Mel-transinfected laboratory line, MGYP2.out (**Figure 3**). In both *w*Mel-infected lines, *Wolbachia* was present in two tissues that are critical in viral infection and dissemination, namely midguts and salivary glands (**Figure 3 A–B & G–H**). *Wolbachia* was also present in brains, although not at high densities which was consistent with levels expected for the *w*Mel strain [9]. Field *w*Mel ovaries appeared highly infected with *Wolbachia* (**Figure 3 D**), indicating the potential for stable transmission of the bacteria to offspring in the wild.

**Figure 4:**
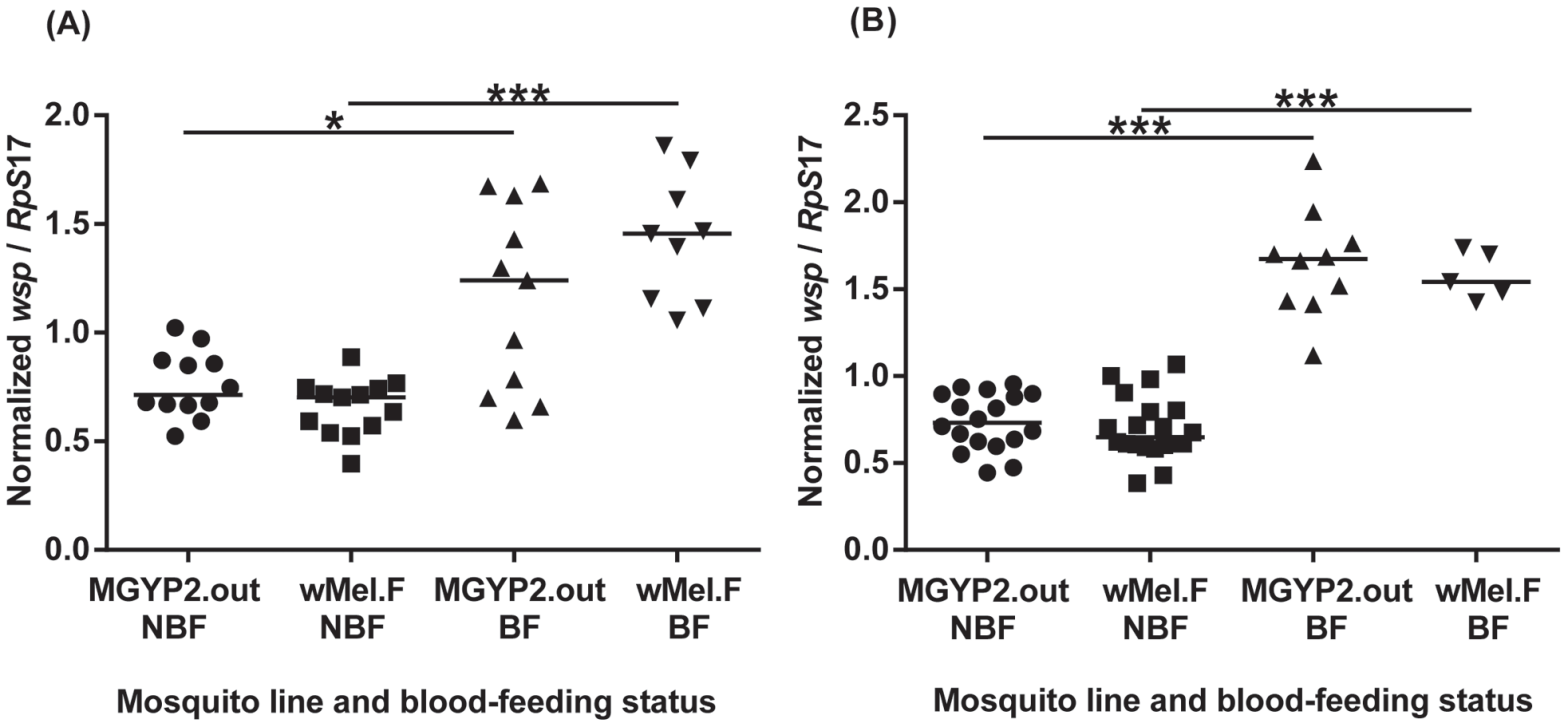
Blood-feeding and *Wolbachia* densities in whole mosquitoes. Outbred laboratory *w*Mel (MGYP2.out) and field-released *w*Mel (*w*Mel.F) *A. aegypti* at 7 (A) and 14 (B) days post blood-feeding (BF) versus non-blood fed (NBF) controls. Bars denote medians. P<0.05 (*), P<0.01 (**), P<0.001 (***). Each point represents an individual mosquito.

**Figure 5:**
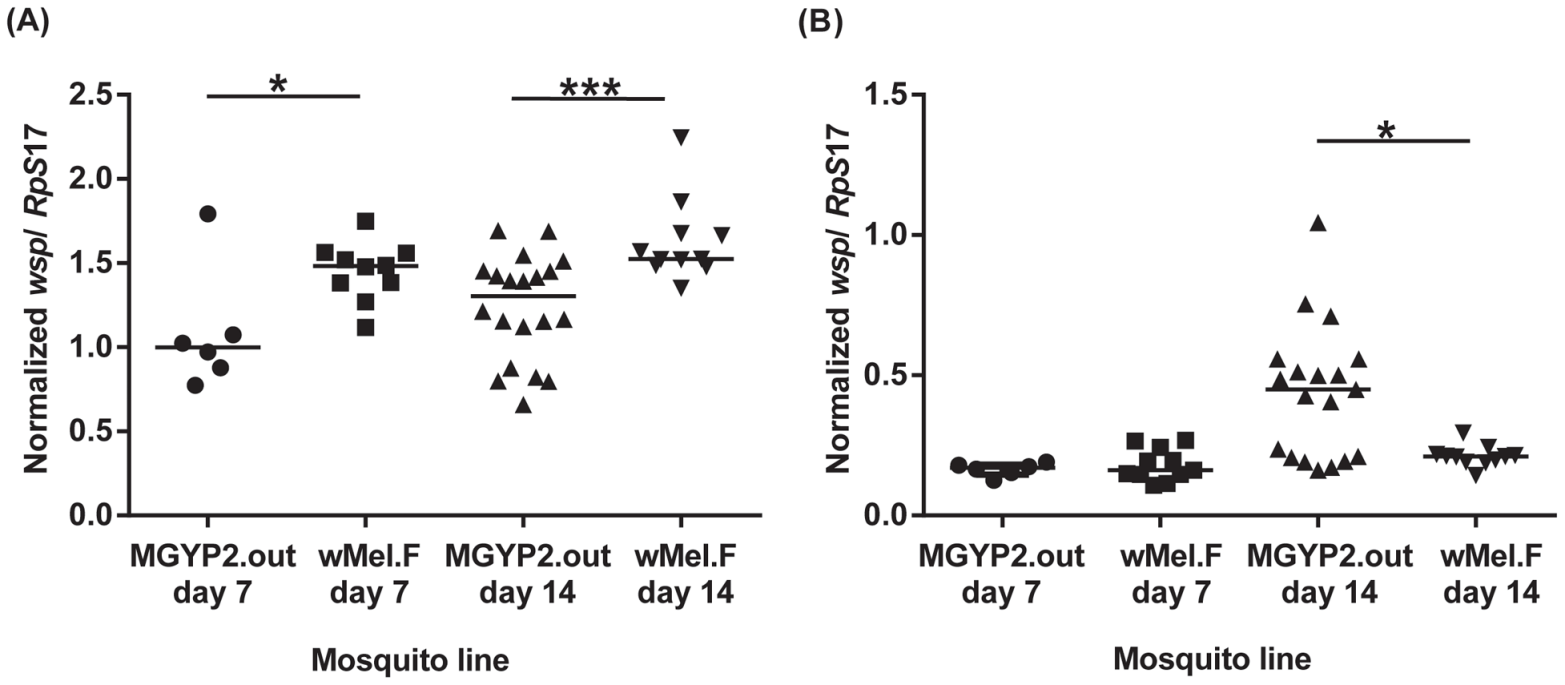
Blood-feeding and *Wolbachia* densities in mosquito heads and bodies. Bodies (A) and heads (B) of outbred laboratory *w*Mel (MGYP2.out) and field-released *w*Mel (*w*Mel.F) *A. aegypti* at days 7 and 14 post blood-feeding. Bars denote medians. P<0.05 (*), P<0.01 (**), P<0.001 (***). Each point represents an individual mosquito.

We also examined whether *Wolbachia* densities change following blood-feeding in field *w*Mel mosquitoes compared to the original MGYP2.out line. By initially looking at whole mosquitoes we found that, by day 7, the density of *w*Mel had increased following blood-feeding in both lines (**Figure 4 A**). A much higher increase in *Wolbachia* density was observed in field *w*Mel mosquitoes versus MGYP2.out (**Figure 4 A**). Median ratios of *wsp* to *Rp*S*17* gene copy numbers increased significantly from 0.714 and 0.702 in non-blood fed *w*Mel.F and MGYP2.out, respectively, to 1.465 and 1.241 in blood-fed *w*Mel.F and MGYP2.out, respectively (**Figure 4 A**). The difference in *Wolbachia* density between blood-fed and non-blood fed mosquitoes persisted at 14 days post feeding (**Figure 4 B**), in the absence of repeat feeds. Median ratios of *wsp* to *Rp*S*17* gene copy numbers were 0.649 and 0.733 in non-blood fed *w*Mel.F and MGYP2.out, respectively, compared to 1.542 and 1.675 in blood-fed *w*Mel.F and MGYP2.out, respectively (**Figure 4 B**). Interestingly, by day 14, *Wolbachia* density continued to increase in blood-fed MGYP2.out and field *w*Mel mosquitoes compared to non-blood fed ones, as indicated by the slightly higher median values of normalized *wsp*/*Rp*S*17* ratios (**Figure 4 B**). Following blood-feeding, increases in *Wolbachia* density in both field and laboratory lines were primarily localized in the bodies rather than heads (**Figure 5**), probably due to the bacteria replicating in ovaries.

## Discussion

Infection of the vector *A. aegypti* with *Wolbachia* has been proposed as a dengue biocontrol method that is environmentally friendly and able to spread unassisted in wild mosquito populations. Release of *w*Mel-infected mosquitoes in north Queensland has indicated that this *Wolbachia* strain can rapidly reach fixation in wild populations [12]. Key to the utility of this biocontrol method is the maintenance of DENV-blocking following mosquito release and in subsequent generations as *Wolbachia* invades wild populations.

Our results indicate that, one year post-release, field *w*Mel mosquitoes show significantly reduced DENV infection and replication compared to wildtype mosquitoes. Strikingly, we found very low infection rates in mosquito heads, indicating that DENV is largely unable to disseminate to the heads in *w*Mel mosquitoes, under the experimental conditions used here. By day 14, in both experiments, *w*Mel mosquitoes displayed dramatically reduced infection rates and viral titers in heads compared to wildtype. Reduced DENV dissemination and transmission rates due to the presence of native *Wolbachia* endosymbionts have also been found in the vector *A. albopictus* [27]. The pattern was observed with a range of virus titers and serotypes (DENV-1 to -3), and using both cell-cultured and viremic human plasma. We did not test for systematic differences in response to these variables here, but work with other viruses has indicated the extent of *Wolbachia*-mediated viral blocking is dependent on virus titer [28].

Our data suggest stability of viral blocking and *Wolbachia* tissue tropism since divergence of field mosquitoes from the parental *w*Mel-transinfected laboratory line MGYP2.out. We did not find statistically significant differences in either dengue infection rates or virus titers between field *w*Mel and MGYP2.out mosquitoes. However, field *w*Mel mosquitoes may be somewhat better at blocking dissemination of DENV-1 but not DENV-2 and DENV-3 compared to MGYP2.out (**Figure 2**). This is because the number of MGYP2.out individuals infected with virus is much higher for DENV-1 than DENV-2 and DENV-3 compared to field mosquitoes. Virus was detected in a higher number of MGYP2.out individuals for DENV-1 strain P307, compared to the other virus strains tested. Additional experiments are needed to determine whether this effect is due to the particular strain or a phenomenon general to the DENV-1 serotype. DENV-2-92T dissemination rates in MGYP2.out were 12.5% several generations after transinfection in earlier work [9] and have stayed a low 7% in our study, at least 10 generations later and with frequent outcrossing of this line (every three generations). This time frame is comparable with that experienced by field mosquitoes, with the maximum number of generations per year in Cairns being 15 and populations persisting throughout the year [29]. MGYP2.out and field *w*Mel-infected mosquitoes have therefore retained the virus blocking phenotype described in [9] that led to the field release of *Wolbachia*-infected mosquitoes. Our results suggest that the virus blocking phenotype induced by *w*Mel may be retained not just over the short term, but also over the medium to longer term.

*Wolbachia* tissue tropism was similar in field and laboratory *w*Mel-infected mosquitoes, with high densities of the bacterium found in the midgut and ovaries. *Wolbachia* was also present in the salivary glands and brains of both mosquito lines, which may contribute to the limited dissemination and replication of DENV observed in heads from the *w*Mel-infected lines. In *Drosophila simulans*, high *Wolbachia* densities in head and midgut have been correlated with interference against *Drosophila* C virus [30].

*Wolbachia* density is critical in modulating transmission fidelity of the bacterium across generations and pathogenicity [19]. *Wolbachia* density changes dynamically in response to environmental variables [31]. We also found that *Wolbachia* density increased following blood-feeding, consistent with other studies that have shown an increase in endosymbiont density in response to high nutrient conditions [32]. *Wolbachia* provides a fitness benefit by modulating iron levels in *D. melanogaster* [33] and responds transcriptionally to iron overload [34]. Increased *Wolbachia* replication is most likely localized to the ovaries, although further work is needed to confirm this. Our results differ, however, from those of [35], who showed a blood-feeding induced reduction in the native endosymbiont *w*Flu in the ovaries of the mosquito *Aedes fluviatilis*. Surprisingly, the increase in *Wolbachia* density was more pronounced in field *w*Mel mosquitoes compared to the laboratory line, although only at day 7 post-infection. The reasons for this difference are unknown but may be related to poor nutrition in the field or other environmental effects. Although mosquitoes were reared in the same environment for one generation, maternal nutritional effects can be detected up to several generations later in insects [36], [37]. Maternal effects due to poor nutrition in the field may influence offspring immune status and the ability to control infection levels, potentially resulting in higher *Wolbachia* densities.

Dynamic changes in *Wolbachia* density following blood-feeding may have implications for vector competence of *w*Mel-infected mosquitoes. The precise mechanism by which *Wolbachia* mediates viral blocking is not known but is positively related to density of the bacterium [13], [14], [38]. If blood-feeding acts to increase *Wolbachia* density and *A. aegypti* feed frequently on human hosts, viral blocking may be greater in field populations than anticipated from laboratory experiments, although further studies are needed to test this hypothesis. In laboratory experiments involving *Drosophila*, the density of *Wolbachia* has been shown to evolve to a level that is non-pathogenic to the fly but the bacteria are still maintained [19], [39]. Understanding selection pressures on *w*Mel-infected mosquitoes in nature will be necessary to predict how *Wolbachia* may evolve over the long term in field-released mosquitoes.

*A. aegypti* infected with *Wolbachia* show reduced replication of other RNA viruses, such as yellow fever [28], chikungunya [8], [28] and West Nile [40] viruses. *Wolbachia*-based biocontrol may therefore have the potential to eliminate transmission of old and emerging arboviruses in addition to DENV. The maintenance of virus blocking in field release mosquitoes is critical to the success of *Wolbachia*-based biocontrol. Our results show that dengue virus blocking and *Wolbachia* density phenotypes have stayed stable in *A. aegypti* infected with *w*Mel, at least 12 months following field release.

## Supporting Information

Figure S1 DENV replication in bodies (A) and heads (B) of wildtype and field-released *w*Mel (*w*Mel.F) *A. aegypti* challenged with three strains (DENV2-92T, DENV1-P249, DENV2-P410), assayed at 7 days post-infection (experiment 1). DENV levels determined using one-step qRT-PCR and expressed as copies per 1 µg of total RNA. Bars denote medians. P<0.05 (*), P<0.01 (**), P<0.001 (***). Each point represents an individual mosquito.
(TIF)

Figure S2 DENV replication in bodies (A) and heads (B) of wildtype, outbred laboratory *w*Mel (MGYP2.out) and field-released *w*Mel (*w*Mel.F) *A. aegypti* challenged with three strains (DENV2-92T, DENV1-P307, DENV3-Cairns08/09), assayed at 7 days post-infection (experiment 2). DENV levels determined using one-step qRT-PCR and expressed as copies per 1 µg of total RNA. Bars denote medians. P<0.05 (*), P<0.01 (**), P<0.001 (***). Each point represents an individual mosquito.
(TIF)
