# Supplementary figures and images for: Limited Dengue Virus Replication in Field-Collected Aedes aegypti Mosquitoes Infected with Wolbachia
Source: PLoS Negl Trop Dis. 2014 Feb 20;8(2):e2688. doi: 10.1371/journal.pntd.0002688 (PMC3930499; doi:10.1371/journal.pntd.0002688)

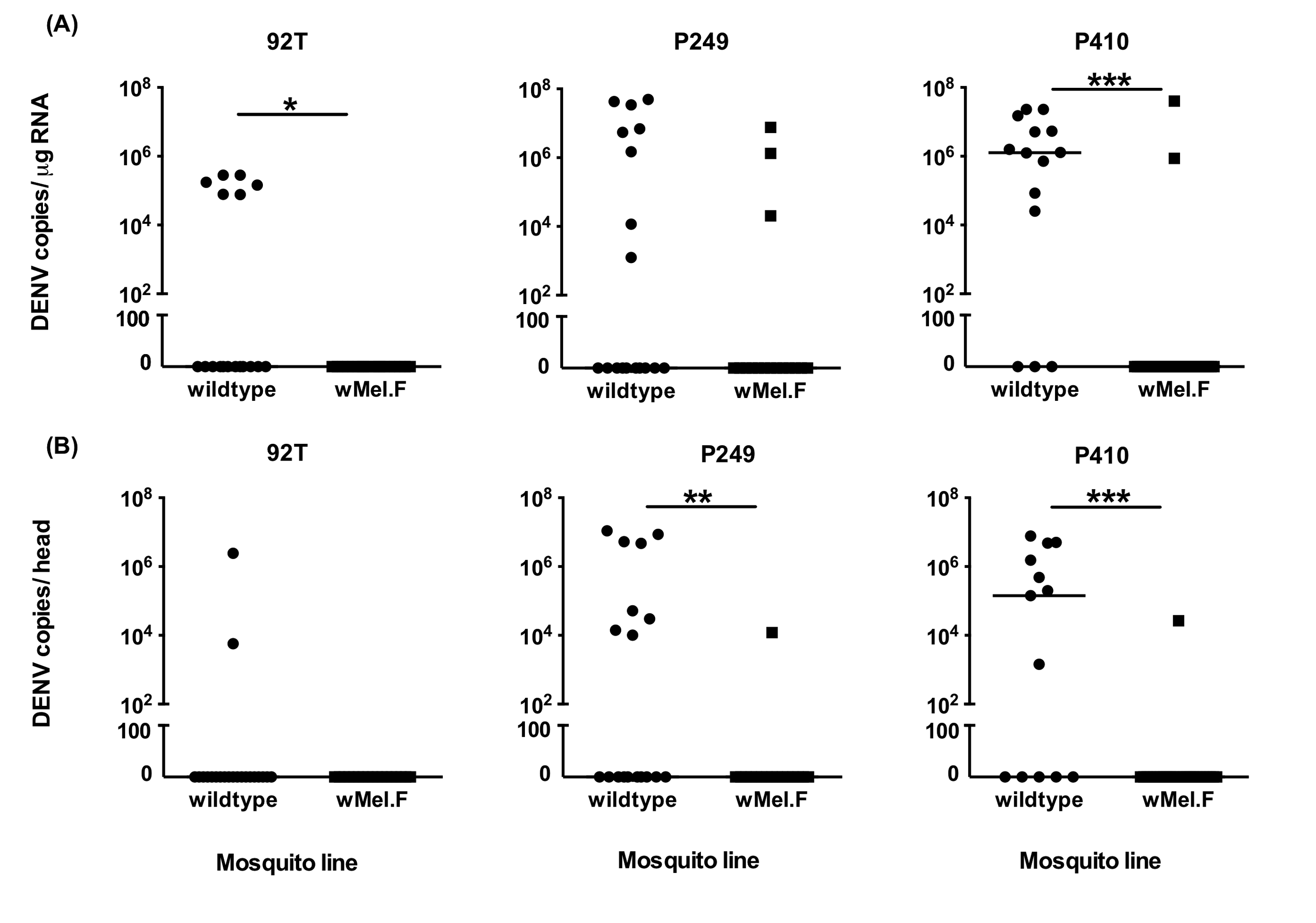

Supplement: Figure S1 — DENV replication in bodies (A) and heads (B) of wildtype and field-released wMel (wMel.F) A. aegypti challenged with three strains (DENV2-92T, DENV1-P249, DENV2-P410), assayed at 7 days post-infection (experiment 1). DENV levels determined using one-step qRT-PCR and expressed as copies per 1 µg of total RNA. Bars denote medians. P<0.05 (*), P<0.01 (**), P<0.001 (***). Each point represents an individual mosquito. (TIF) [file pntd.0002688.s001.tif]

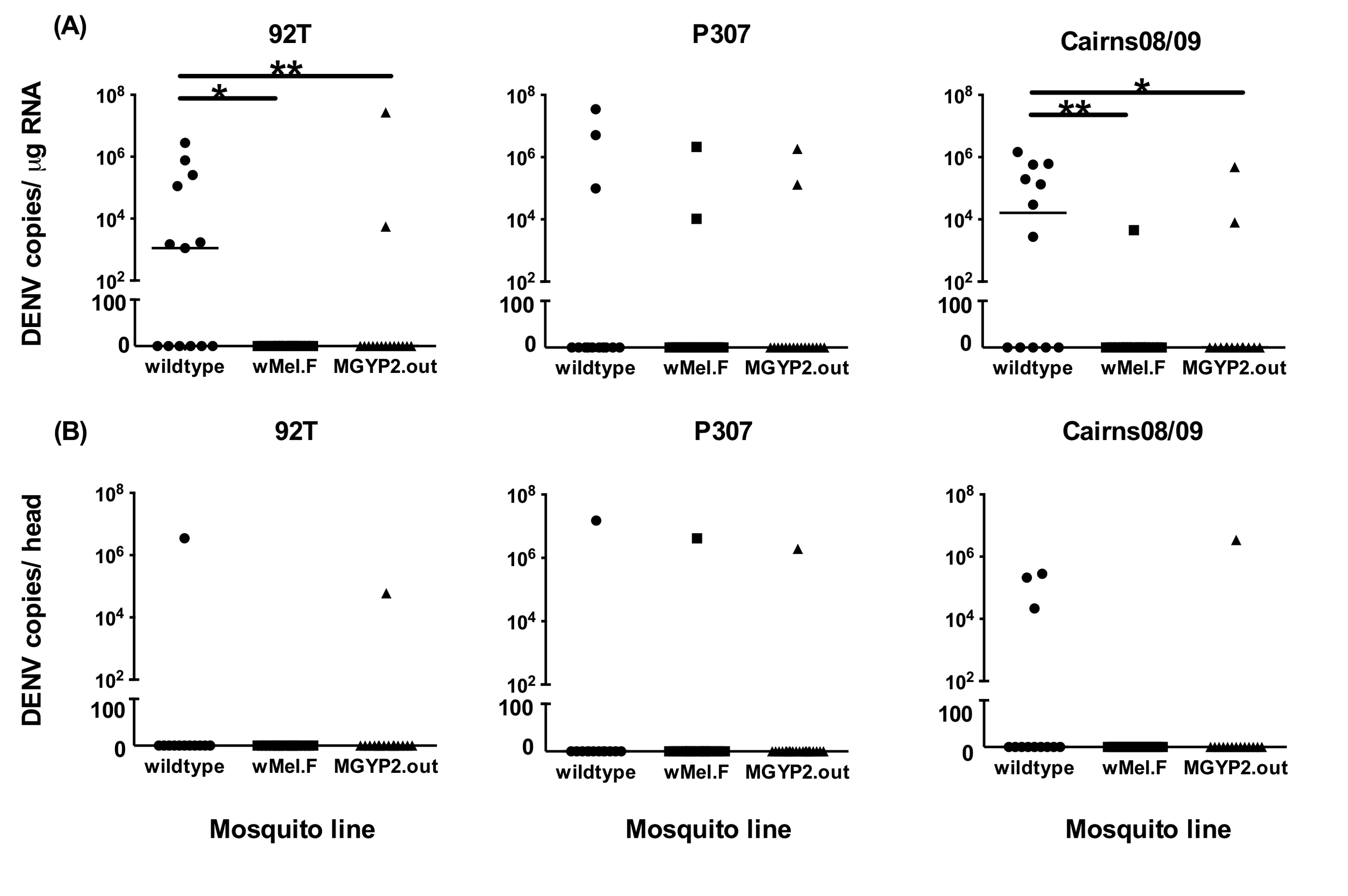

Supplement: Figure S2 — DENV replication in bodies (A) and heads (B) of wildtype, outbred laboratory wMel (MGYP2.out) and field-released wMel (wMel.F) A. aegypti challenged with three strains (DENV2-92T, DENV1-P307, DENV3-Cairns08/09), assayed at 7 days post-infection (experiment 2). DENV levels determined using one-step qRT-PCR and expressed as copies per 1 µg of total RNA. Bars denote medians. P<0.05 (*), P<0.01 (**), P<0.001 (***). Each point represents an individual mosquito. (TIF) [file pntd.0002688.s002.tif]
